# The Role of BRG1 in Antioxidant and Redox Signaling

**DOI:** 10.1155/2020/6095673

**Published:** 2020-09-14

**Authors:** Shilong You, Ying Zhang, Jiaqi Xu, Hao Qian, Shaojun Wu, Boquan Wu, Saien Lu, Yingxian Sun, Naijin Zhang

**Affiliations:** Department of Cardiology, The First Hospital of China Medical University, Shenyang, Liaoning, China

## Abstract

Redox homeostasis is regulated by critical molecules that modulate antioxidant and redox signaling (ARS) within the cell. Imbalances among these molecules can lead to oxidative stress and damage to cell functions, causing a variety of diseases. Brahma-related gene 1 (BRG1), also known as SMARCA4, is the central ATPase catalytic subunit of the switch/sucrose nonfermentable (SWI/SNF) chromatin remodeling complex, which plays a core role in DNA replication, repair, recombination, and transcriptional regulation. Numerous recent studies show that BRG1 is involved in the regulation of various cellular processes associated with ARS. BRG1, as a major factor in chromatin remodeling, is essential for the repair of oxidative stress-induced DNA damage and the activation of antioxidant genes under oxidative stress. Consequently, a comprehensive understanding of the roles of BRG1 in redox homeostasis is crucial to understand the normal functioning as well as pathological mechanisms. In this review, we summarized and discussed the role of BRG1 in the regulation of ARS.

## 1. Introduction

Brahma-related gene 1 (BRG1), also known as SMARCA4, is the central catalytic ATPase of the switch/sucrose nonfermentable (SWI/SNF) chromatin remodeling complex, which alters the structure of reconstituted chromatin particles in an ATP-dependent manner and makes genomic regions more accessible to transcription factors and the transcription machinery [1]. As a major factor in chromatin remodeling, BRG1 plays a pivotal role in DNA replication, repair, recombination, and transcriptional regulation by interacting with various nuclear proteins, including nuclear receptors, transcription factors, and chromatin modifying enzymes [2, 3]. Consequently, BRG1 is involved in a range of cellular processes, including cell proliferation, apoptosis, and differentiation [4–6], and is involved in a diversity of diseases, such as cancer [7], liver fibrosis [8] and heart disease [9]. Accumulating evidence indicates that BRG1-mediated chromatin remodeling is essential for the repair of oxidative stress-induced DNA damage and the activation of antioxidant genes under oxidative stress [10–13]. In view of the importance of BRG1 in oxidative stress, in this review, we critically discuss the potential role of BRG1 in redox regulation, oxidative stress, and reactive oxygen species- (ROS-) induced disease.

## 2. BRG1

The *BRG1* gene is located in chromosomal region 19p13.2 [14]. A gene enrichment analysis of the regions of the genome occupied by BRG1 showed that BRG1 occupied the promoter regions of the hypoxia-inducible factor (HIF)2*α* transcription factor and of metabolic regulators in several key pathways including the glycolytic pathway [15]. Interestingly, this study also found that oxidative stress-induced BRG1 could bind to the promoter of the antioxidant defense gene and induce its transcription, thus protecting cells from oxidative damage. Sena et al. further reported that BRG1 can promote the expression of *HIF1α* and *HIF2α* genes and promote hypoxic induction of a subset of HIF1 and HIF2 target genes to regulate the hypoxia response [16]. HIF1*α*, HIF2*α*, and many other key metabolic regulators are major regulators of survival pathways activated by various cellular stresses (such as hypoxia) [17, 18]; therefore, it is reasonable to assume that BRG1 also plays a key role in regulating oxidative stress. Moreover, recent studies showed that BRG1 overexpression mitigated hypoxia-induced cell damage, while BRG1 suppression contributed to hypoxia-induced cell damage [19]. Taken together, these studies suggest that BRG1 is involved in oxidative stress and may have an antioxidant effect.

## 3. BRG1 and Oxidative Stress

Redox species, the primary forms of which include ROS and reactive nitrogen species (RNS), have dual roles in living systems, having beneficial or deleterious effects [20]. Normal levels of redox species are essential to trigger many important reactions and signaling cascades [21]. However, when produced in excess or combined with other redox species, they can have serious deleterious effects on cellular function (reviewed in references [22, 23]), which can be counteracted by enzymatic and nonenzymatic antioxidant systems. The delicate balance between the beneficial and deleterious effects of redox species, known as redox homeostasis, is achieved through a series of regulatory mechanisms and is an important aspect of healthy organisms [20]. Imbalanced redox homeostasis caused by inappropriate biochemical reactions in cells and/or external factors may result in oxidative stress [24], which is a pivotal cause of many diseases, such as cancer [25], cardiovascular disease [26], neurodegenerative diseases [27], and diabetes [28].

Numerous studies show that oxidative stress can induce DNA damage, leading to single- or double-strand breaks, which can be fatal to cells if not repaired [29]. The most common lesion generated by intracellular oxidative stress is 8-hydroxydeoxy guanosine (8-oxodG) [30]. During oxidative stress, the processing and repair of 8-oxodG in nucleosomes requires ATP-dependent chromatin remodeling [10]. BRG1, as a major factor in chromatin remodeling, takes part in protecting against DNA damage induced by oxidative stress and regulating redox homeostasis. Moreover, the formation of BRG1-promoter complexes in response to oxidative stress is essential to protect antioxidant gene promoters from oxidative damage [15]. The specific mechanisms by which BRG1 chromatin remodeling complexes mediate these redox and antioxidant signals are a current focus of research. Therefore, below, we discuss the diverse ways that BRG1 and ARS molecules associate and how this knowledge can be applied to the treatment of related diseases.

## 4. BRG1 in Oxidative Stress Signaling

Accumulating evidence associates ARS events with the BRG1 chromatin remodeling complex. The relationship between ARS events and BRG1 is complex and multifaceted, involving multiple mechanisms that regulate antioxidant and redox signaling pathways and that affect redox homeostasis. Below, we categorically describe the regulation of BRG1 in redox signaling and its role in oxidative stress-mediated diseases.

### 4.1. BRG1 in Keap1/Nrf2 Signaling

One of the most studied transcription factors activated by oxidative stress is nuclear factor E2-related factor 2 (Nrf2), which is responsible for inducing the expression of several antioxidant defense genes and is the main regulator of cellular defense against oxidative stress [31]. In response to oxidative stress, Nrf2 is activated by dissociation from the inhibitor Kelch-like ECH-associated protein-1 (Keap1) and transferred into the nucleus, where it binds to the antioxidant reaction element (ARE), thereby promoting the expression of antioxidant genes, such as heme oxygenase-1 (HO-1) and signal transducer and activator of transcription 3 (STAT3) [32].

#### 4.1.1. BRG1 in Keap1/Nrf2/HO-1 Signaling

Early studies showed that Nrf2 binding to AREs requires BRG1 recruitment to induce *HO-1* gene activation. Hyperphosphorylation of BRG1 prevents HO1-induced oxidative damage and is considered to be a key mechanism of darinaparsin-induced apoptosis [33]. Upon oxidative stress, BRG1 interacts with Nrf2 to activate *HO-1* gene expression by promoting the formation of Z-DNA and subsequently recruiting RNA polymerase II to the promoter of *HO-1* [34, 35]. Therefore, activation of HO-1 by the interaction between BRG1 and Nrf2 may be a key regulatory checkpoint in the regulation of disease states caused by oxidative stress.

BRG1-mediated Nrf2/HO-1 signaling has been reported in diverse pathological processes. The expression of BRG1 was significantly decreased in the myocardium of diabetic rats, which was at least partly related to the decreased expression of *HO-1* and impaired diastolic function [36]. Similarly, the depletion of BRG1 caused by high blood sugar significantly blocked the cardioprotective effects of sevoflurane postconditioning, or emulsified isoflurane postconditioning, because of impaired Nrf2/HO-1 signaling [37, 38]. Furthermore, adiponectin promotes *HO-1* expression by simultaneously activating Nrf2 and BRG1, thereby inhibiting hyperglycemic-induced oxidative stress, myocardial cell apoptosis, cardiac hypertrophy, and cardiac dysfunction [39]. These studies may provide a new way to treat diabetic cardiomyopathy.

BRG1-mediated Nrf2/HO-1 transcriptional activation is important for oxidative stress injury induced by ischemia/reperfusion (I/R) or hypoxia/reoxygenation (H/R). Propofol alleviates oxidative stress in anoxia/reoxygenated hepatocytes through the lncRNA-TUG1/BRG1 pathway [40]. Knockdown of BRG1 inhibited *HO-1* expression, thereby weakening the protective effect of propofol postconditioning on hepatic ischemia/reperfusion injury (HIRI) [41]. Consistent with these results, overexpression of BRG1 alleviates hepatic I/R injury by activating the Nrf2/HO-1 signaling pathway [42]. Furthermore, under BRG1 overexpression, the upregulation and nuclear translocation of Nrf2 alleviates acute lung injury caused by hepatic I/R via activation of the antioxidant enzyme system, including NAD (P) H quinone dehydrogenase 1 (NQO1), superoxide dismutase (SOD), glutamate-cysteine ligase catalytic subunit (GCLC), and glutathione S-transferase alpha 1 (GST*α*1) [43]. This suggests that BRG1 may also play a key role in promoting the activation and expression of antioxidant enzymes.

Deng et al. found that under oxidative stress, the number of damaged and dead neurons in BRG1 knockout (KO) mice increased significantly, which may be related to the regulation of NR2B-NR2A by BRG1 [44]. Overexpression of BRG1 increases the activity of neurons, weakens apoptosis, and reduces the production of ROS, thus producing a protective effect on oxygen-glucose deprivation/reoxygenation- (OGD/R-) induced injury [45]. Additionally, further evidence indicates that inhibition of miR-144-3p or miR-199a-5p can alleviate OGD/R-induced neuronal injury by upregulating BRG1 to activate Nrf2/HO-1 signaling [46, 47].

**Figure 1:**
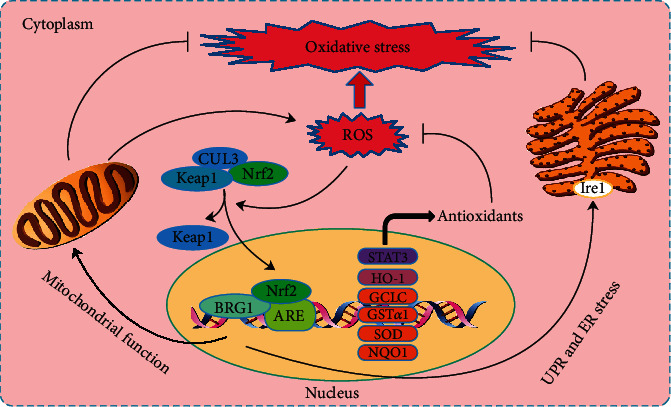
The figure illustrates interaction of BRG1 with Nrf2 that regulates the expression of several antioxidant genes. Under oxidative stress, the Keap1-CUL3 ubiquitination system is disrupted, resulting in the dissociation of Nrf2 and its translocation into the nucleus where it binds ARE, thereby promoting the expression of antioxidant genes. In response to oxidative stress, BRG1 also has a protective effect on mitochondrial function and regulates the UPR and ER stress by maintaining the basal level of Ire1 activity. ARE: antioxidant reaction element; ER: endoplasmic reticulum; CUL3: cullin 3; Keap1: Kelch-like ECH-associated protein-1; Nrf2: nuclear factor E2-related factor 2; UPR: unfolded protein response.

From the above studies, it can be inferred that BRG1 can protect tissues from I/R-, H/R-, or OGD/R-induced oxidative injury through Keap1/Nrf2/HO-1 signal transduction (Figure 1). BRG1 is therefore a potential target for the treatment of I/R-, H/R-, or OGD/R-induced injury. Paradoxically, Naito et al. suggested that BRG1, in ischemic stress, induces tumor necrosis factor-*α* (TNF-*α*) expression (TNF-*α* promotes the production of ROS, reviewed in reference [48]) in renal epithelial cells, exacerbating renal injury [49]. Furthermore, Liu et al. reported that BRG1 regulated endodermal IL-33, which aggravated renal injury and fibrosis induced by ischemia-reperfusion in mice [50]. Therefore, the role of BRG1 in ischemia-reperfusion is controversial and deserves further study.

#### 4.1.2. BRG1 in Keap1/Nrf2/STAT3 Signaling

Myocardial STAT3 is a crucial transcription factor in the SAFE pathway (i.e., Janus kinase (JAK)2/STAT3 signaling cascade), especially during myocardial ischemia reperfusion injury [51]. STAT3 restores the expression of endothelial nitric oxide synthase (eNOS) and maintains the transcription of eNOS in endothelial cells to cope with hypoxia injury [52]. Upon exposure to oxidative stress, BRG1 interacts with Nrf2 and subsequently participates in Nrf2-mediated STAT3 gene activation [38]. BRG1 also provides protection against ROS-induced cardiomyopathy by activating STAT3 [53]. Furthermore, a study by Wang et al. showed that the cardiac protective effect of emulsified isoflurane postconditioning was lost in diabetic rats because of impaired Nrf2/STAT3 signal transduction [38]. These results indicate that BRG1 plays a vital role in antioxidant damage through Keap1/Nrf2/STAT3 signaling (Figure 1).

### 4.2. BRG1 in Other ROS-Mediated Signaling Pathways

In addition to the Keap1/Nrf2 signaling pathway, BRG1 is involved in a number of other ROS-mediated pathways that are closely related to oxidative stress.

#### 4.2.1. BRG1 in NF-*κ*B and p65 Signaling

**Figure 2:**
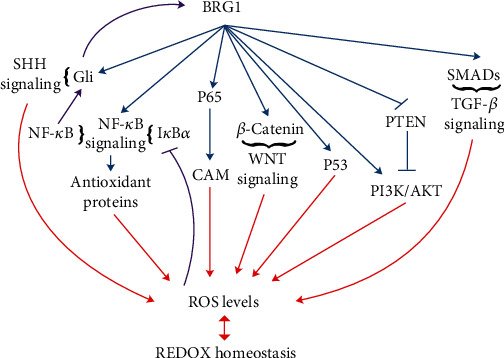
Pathways that are closely related to ROS levels implicated in BRG1 function in oxidative homeostasis. The summary diagram is simplified (see text for details).

There is a strong mutual relationship between ROS and nuclear factor kappa B (NF-*κ*B) signaling. ROS affects activation of NF-*κ*B signaling mainly by inhibiting the phosphorylation of I*κ*B*α*. In turn, NF-*κ*B signaling can also affect ROS levels by upregulating the expression of antioxidant proteins, such as SOD and glutathione peroxidase (GPX) [54, 55]. Moreover, p65 exacerbates hypoxia stress-mediated endothelial cells [56]. Under hypoxia, p65 at least partially induces endothelial dysfunction by activating a series of cell adhesion molecule (CAM) genes [57]. Fang et al. showed that BRG1 and Brahma (BRM) interact with NF-*κ*B/p65 and fine-tune the binding of p65 to target promoters, which indicates a strong correlation between BRG1 and oxidative stress signals related to ROS [58] (Figure 2).

#### 4.2.2. BRG1 in p53 and PTEN/PI3K-AKT Signaling

p53 is the main modulator of a cell's response to various stresses, including oxidative stress. Under physiological conditions or exposure to transient and mild oxidative stress, p53 has an antioxidant function and contributes to maintaining low levels of ROS (reviewed in reference [59]). Phosphatase and tensin homolog (PTEN)/phosphatidylinositol 3-kinase (PI3K)-AKT signaling also mediates ROS damage to cells [60, 61]. Notably, BRG1 has a direct effect on p53 [62] and may inhibit the expression of PTEN at mRNA and protein levels, thereby upregulating the PI3K-AKT signaling pathway [63, 64] (Figure 2). These results further support the important role of BRG1 in the regulation of oxidative stress.

#### 4.2.3. BRG1 in SHH Signaling

The Sonic hedgehog (SHH) signaling pathway protects cell homeostasis by regulating antioxidant defense mechanisms and maintaining mitochondrial dynamics to resist oxidative stress [65]. BRG1 is required for the transcriptional activation of *Gli* in the SHH pathway [66]. Moreover, SHH, activated by BRG1-recruited NF-*κ*B, can in turn activate BRG1 expression through Gli to sustain a positive feedback loop [67] (Figure 2). The relationship between BRG1 and SHH signaling may be an important basis for BRG1 to participate in the regulation of redox homeostasis.

#### 4.2.4. BRG1 in TGF-*β* and WNT Signaling

The level of ROS is closely related to the transforming growth factor beta (TGF-*β*) [68] and WNT signaling pathways [69]. Importantly, BRG1 not only enhances TGF-*β* signaling as a cofactor of SMADs [70], but also participates in the WNT pathway by controlling the bioavailability of signaling molecules (such as ligands, receptors, and signal adapters) and by directly activating the target gene, *β-catenin* [71, 72] (Figure 2). Therefore, BRG1 may be involved in the management of redox signals by regulating TGF-*β* signaling and WNT signaling.

In short, BRG1 can affect intracellular ROS levels by participating in a number of ROS-related signaling pathways. However, the specific mechanism by which BRG1 influences the redox signal through these pathways needs to be clarified. Further investigation is expected to reveal many new roles of BRG1 in redox homeostasis.

### 4.3. BRG1 and Mitochondrial Function

It has been established that more than 90% of ROS are generated by mitochondria in eukaryotic cells [73]. Mitochondrial dysfunction is one of the main reasons for the accumulation of ROS and oxidative-damaged proteins in cells [74]. BRG1 takes part in mitochondrial oxidative pathways and therefore affects the production of ROS. During the heat shock response, BRG1 recruited by heat shock factor 1 (HSF1) helps to maintain mitochondrial membrane potential and cell survival by activating transcription of the gene encoding mitochondrial chaperone, such as heat-shock protein (HSP) 60, HSP10, and mtHSP70 [75]. BRG1 and BRM can maintain cardiomyocyte homeostasis *in vivo* by regulating mitochondrial dynamics and mitotic phagocytosis. In the BRG1-BRM double-knockout mouse heart, mitochondria were small and fragmented, with a decrease in both number and size [76].

Increased mitochondrial respiration was observed in BRG1-deficient tumor cells. This was because the expression of several important genes in the oxidative phosphorylation (OXPHOS) pathway increased, including the master mitochondrial biogenesis coactivator, PGC1-*α*, mitochondrial ATP synthase F0 complex subunit (ATP5L), and oxidative stress response genes, such as glutathione S-transferase omega 7 (GSTO7) and GSTO1. The level of several metabolites that play a key role in the pentose-phosphate pathway (PPP) was also elevated [77]. It is worth noting that, under hypoxic conditions, mitochondrial complex I (CI) dysfunction causes high levels of ROS, triggering a signal transduction pathway involving BRG1 downregulation [78]. Furthermore, deletion of BRG1 caused a significant upregulation in the expression of genes related to mitochondrial degradation, which may lead to an imbalance in redox homeostasis [79].

In general, these studies indicate that BRG1 plays an active role in protecting mitochondrial function and that BRG1 may protect organisms from metabolic damage through mechanisms that depend on oxidative stress (Figure 1).

### 4.4. BRG1 and ER Stress

Accumulation of unfolded and misfolded proteins or excessive protein transport in the endoplasmic reticulum (ER) can cause ER stress and trigger the unfolded protein response (UPR). UPR is modulated by the ER and the redox system in the ER [80]. In the presence of ER stress, the UPR uses evolutionarily conserved signaling pathways to restore normal cell function and inhibit apoptosis, which are necessary and sufficient to alleviate protein aggregation and ER stress [81]. BRG1 maintains the basal level of inositol-requiring enzyme 1 (Ire1) activity by reducing oxidative stress in the ER network, which promotes UPR regulation and the clearance of cytoplasmic protein aggregates to maintain ER homeostasis [82]. These data indicate that BRG1 plays a critical role in oxidative stress by maintaining ER homeostasis (Figure 1).

### 4.5. BRG1 and Autophagy

**Figure 3:**
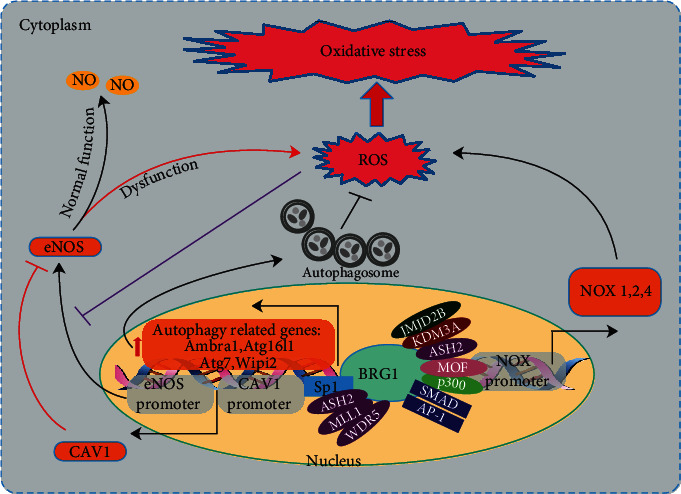
The figure shows the transcriptional activation of BRG1 on a variety of ARS-related genes, including NOX (1,2,4), eNOS, CAV1, and autophagy-related genes. Interestingly, BRG1 not only restores the expression of eNOS to resist oxidative stress but also aggravates oxidative stress by activating CAV1 to limit eNOS activity and NO bioavailability. CAV1: caveolin-1; eNOS: endothelial nitric oxide synthase; NOX: NADPH oxidase complex.

Autophagy, a lysosomal degradation pathway, is responsible for the removal of aggregation-prone proteins, the disposal of excessive or damaged organelles, and the renewal of long-lived proteins, to achieve the metabolic needs of the cell and the turnover of certain organelles [83]. Autophagy is believed to be the main mechanism for removing oxidized proteins from cells [84], and impaired autophagy causes oxidative stress [85]. The transcription of central autophagy regulatory genes, such as *Atg16l1*, *Ambra1*, *Atg7*, and *Wipi2*, was directly modulated by BRG1. Autophagy defects in BRG1-deficient intestinal epithelial cells (IECs) lead to overproduction of ROS, resulting in a defect of barrier integrity [86]. Therefore, it is reasonable to believe that BRG1 participates in redox signaling by regulating autophagy (Figure 3).

### 4.6. BRG1 and NOX

ROS play a wide range of physiological and pathophysiological roles in various processes. The production of ROS is catalyzed by a group of specialized enzyme families, of which the NADPH oxidase (NOX) family is the most studied [87, 88]. Since its discovery in the late 1970s [89], NOX—as a main source of ROS production—has been demonstrated to be involved in a variety of ROS-mediated physiological and pathophysiological processes [90].

NOX4-catalyzed ROS generation in endothelial cells promotes tissue fibrosis by directing endothelial cells toward a myofibroblast-like phenotype in a process known as endothelial-mesenchymal transition [91]. A very recent study by Li et al. showed that BRG1 interacts with KDM3A to activate NOX (1, 2, 4) transcription in endothelial cells. Endothelial-derived ROS, generated by increased NOX expression, may promote cardiac ischemia-reperfusion injury [92]. Similarly, Liu et al. reported that BRG1 relied on lysine acetyltransferase 8 (KAT8) to activate the transcription of NOX genes (*NOX1*, *NOX2*, and *NOX4*), to promote intracellular ROS production in a mouse model of nonalcoholic steatohepatitis [93]. Moreover, according to Li et al., BRG1 interacts with SMAD3 and AP-1 to upregulate histone demethylase JMJD2B, histone acetyltransferase p300, and ASH2 (a key regulatory subunit of the H3K4 methyltransferase complex), which mediates TGF-*β*-induced transcription of NOX4 in endothelial cells and stimulates the production of ROS, to promote endothelial-mesenchymal transition and liver fibrosis [94].

In general, BRG1 regulates NOX transcription to promote ROS production and aggravate oxidative stress-induced damage. However, these observations are contradictory to a series of findings that suggest BRG1 protects against oxidative damage. It is of high interest to explain the dual role of BRG1 in oxidative stress.

### 4.7. BRG1 and eNOS

Under normal physiological conditions, eNOS is the main source of nitric oxide (NO) in the vascular system [95]. However, instead of producing NO, dysfunctional eNOS can produce a superoxide anion radical (O^2−^), resulting in decreased NO bioavailability and increased oxidative stress, causing and exacerbating endothelial dysfunction [96, 97]. Fish et al. reported that BRG1 helped to restore eNOS expression during the anoxia/reoxygenation cycle by preventing the ejection of acetylated H3 and H4 histones on eNOS promoters in endothelial cells [52]. It can be inferred that BRG1 protected endothelial function under oxidative stress by restoring eNOS expression. In contrast, Shao et al. found that endothelial BRG1 limited eNOS activity and NO bioavailability by activating caveolin-1 (*CAV1*) transcription, contributing to thioacetamide-induced liver fibrosis in mice [98] (Figure 3). Hence, the exact effect of BRG1 on eNOS expression and activity needs further study.

### 4.8. BRG1 and MTs

Metallothioneins (MTs), encoded by *MT* genes, are a group of low molecular weight, cysteine-rich metal-binding proteins that act as antioxidants to prevent DNA damage and apoptosis by maintaining intracellular metal homeostasis and redox balance [99, 100]. Through a chromatin immunoprecipitation assay, Datta et al. revealed that ATP-dependent chromatin remodeling of BRG1 produced a significant inhibitory effect on *MT-1* promoter activity in mouse lymphosarcoma cells [101]. Therefore, BRG1 may play a key role in the regulation of antioxidant molecules, even if not by direct regulation of BRG1 itself.

## 5. Conclusion

**Figure 4:**
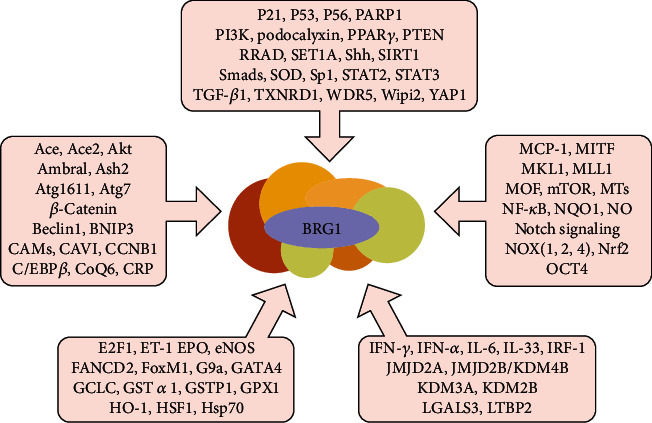
Molecular associations of BRG1 with ARS-related targets. Schematic representation shows a wide variety of ARS-related targets regulated with BRG1. Ace: angiotensin-converting enzymes; CAMs: cell adhesion molecules; CAV1: caveolin-1; CCNB1: cyclin B1; C/EBP*β*: CCAAT-enhancer-binding protein; CRP: C-reactive protein; ET-1: endothelin-1; EPO: erythropoietin; FoxM1: forkhead box M1; HSF1: heat shock factor 1; LGALS3: Galectin-3; LTBP2: latent TGF-*β*-binding protein 2; MCP-1: monocyte chemoattractant protein 1; MITF: microphthalmia-associated transcription factor; MKL1: megakaryocytic leukemia 1; MOF: males absent on the first; MTs: metallothioneins; PPAR*γ*: peroxisome proliferators-activated receptor *γ*; RRAD: Ras-related associated with diabetes; SHH: Sonic hedgehog; TGF-*β*1: transforming growth factor beta 1.

BRG1 plays an important role in redox regulation and regulating cellular homeostasis by establishing specific gene expression patterns and maintaining the transcriptional state. The maintenance of redox homeostasis is required for various feedback mechanisms, mainly related to transcriptional modulation, to function at different levels. As discussed above and summarized in Figure 4, BRG1 regulates many of the pivotal genes and molecules related to redox homeostasis and oxidative stress. The majority of studies show that BRG1 protects cells from oxidative stress damage by promoting the formation of antioxidants, or suppressing the production of ROS, or both. However, a minority of studies show that BRG1 aggravates oxidative stress damage by inducing ROS generation. Overall, the obvious protective effects of BRG1 against oxidative stress damage support the theory that BRG1 synergistically maintains cellular homeostasis via distinct mechanisms. This may provide the mechanistic basis for the development and discovery of antioxidants and BRG1 regulators for the management of oxidative stress-related diseases. Tables 1 and 2 summarize BRG1 regulators and their known actions. Further in-depth studies are required to identify and clarify the exact roles and the dual function of BRG1 in regulating redox homeostasis. Further research addressing these issues, with focus on BRG1 as a drug target, may provide therapeutic strategies for the treatment of cancer, ischemia-reperfusion injury, and other redox-related diseases.

## Figures and Tables

**Table 1:** The nucleic acid and protein modulators of BRG1. This list summarizes the current research on the nucleic acid and protein modulators of BRG1. ARE: antioxidant reaction element; BAF: BRG1/BRM-associated factor; BRG1: Brahma-related gene-1; BRM: Brahma; CK2: casein kinase 2; HO-1: heme oxygenase 1; lncRNA: long noncoding RNA; MiR: microRNA; ncRNA: noncoding RNA; NKTCL: natural killer/T-cell lymphoma; Nrf2: nuclear factor E2-related factor 2; OGD/R: oxygen-glucose deprivation/reoxygenation; PPAR*γ*: peroxisome proliferators-activated receptor *γ*; SWI/SNF: switch/sucrose nonfermentable; UCA1: urothelial carcinoma-associated 1.

Modulators	Known actions	Ref.
Nucleic acids		
Btr	Under anaerobic conditions, Btr can increase the expression of BRG1 in *E. coli* by three times.	[102]
LncRNA Evf2	Evf2 directly inhibits BRG1 ATPase and chromatin remodeling activities.	[103]
LncRNA UCA1	UCA1, as a suppressor of BRG1, promotes bladder cancer cell proliferation by inhibiting BRG1.	[104]
MiR-101, miR-199, and miR-155	BRG1 expression is controlled by miR-101, miR-199, and miR-155 through binding to 3′UTRs.	[105]
MiR-155	There is a negative correlation between miR-155 level and BRG1 in normal NK, as well as two NKTCL cell lines and the MOLT4 cell line.	[106, 107]
MiR-139-5p	MiR-139-5p promotes apoptosis and suppresses proliferation of human airway smooth muscle cells by decreasing the BRG1.	[108, 109]
MiR-144-3p	MiR-144-3p promotes OGD/R-induced neuronal injury by negatively regulating BRG1/Nrf2/ARE signaling.	[46]
MiR-199a-5p	Downregulation of miR-199a-5p can protect neurons from OGD/R-induced neuron damage by upregulating BRG1 to activate Nrf2/HO-1 signaling.	[47] [110]
MiR-21	BRG1 is a direct target of miR-21.	[111]
MiR-221/222	BRG1 is the most likely target affected by miR-221/222 during LPS tolerance, and increased expression of miR-221/222 reduces BRG1 expression.	[112]
MiR-206	BRG1 may be an important gene target of miR-206 during carcinogenesis and osteogenesis.	[113]
MiR-302	MiR-302 binds the 3′UTRs and directly regulates the BRG1 complex subunits BAF53a and BAF170.	[114]
MiR-99a	BRG1 is positively regulated by miR-99a and is involved in hypoxia-induced cell injuries in H9C2 cells.	[19]
NcRNA Xist	Xist binding inhibits BRG1's nucleosome-remodeling activity and results in expulsion of the SWI/SNF complex from the Xi.	[115]
Proteins		
Angiotensin II	Expression of BRG1 is increased in vitro when cardiomyocytes are stimulated with angiotensin II or a *β*-adrenergic agonist.	[116]
Calcineurin	Calcineurin (Cn) regulates the ability of BRG1 and other SWI/SNF enzyme subunits to stably associate with myogenic promoters during differentiation.	[117]
Camk2a	Camk2a-Cre-mediated conditional deletion of BRG1 leads to perinatal hydrocephalus.	[118]
CK2	CK2-mediated phosphorylation of BRG1 regulates myoblast proliferation.	[119]
Cdx	Cdx transcription factors regulate target gene expression, in part, through recruitment of BRG1-associated SWI/SNF chromatin remodeling activity.	[120]
Gcn5	In vivo and in vitro, the Snf2 subunit of the SWI/SNF complex is acetylated directly by the Gcn5-containing complexes.	[121]
NRG1	BRG1 expression is inhibited in the NRG1*Δ* mutant and BRG1's induction was blocked by overexpression of NRG1.	[122]
p63	P63 directly modulates the expression of BRG1.	[123]
PPAR*γ*	PPAR*γ* activation-mediated inhibition of BRG1 activity through NF-*κ*B pathway.	[124]
SCF FBW7	SCF FBW7-mediated degradation of BRG1 inhibits gastric cancer metastasis.	[125]
SRG3	SRG3 protects the major components of the SWI/SNF complex from proteasomal degradation by interacting directly with them.	[126]

**Table 2:** The chemical modulators of BRG1. This list summarizes the current research on the chemical modulators of BRG1. BRG1: Brahma-related gene-1; BRM: Brahma; CDK: cyclin-dependent kinase; ENT: entinostat; HO-1: heme oxygenase 1; H/R: hypoxia/reoxygenation; IsoPostC: isoflurane postconditioning; NAC: N-acetylcysteine; Nrf2: nuclear factor E2-related factor 2; PPC: propofol postconditioning; SPostC: sevoflurane postconditioning; STAT3: signal transducer and activator of transcription 3; SWI/SNF: switch/sucrose nonfermentable.

Modulators	Known actions	Ref.
Chemicals		
17*β*-estradiol	17*β*-estradiol antagonizes both the expression and activity of BRG1/BRM.	[58]
Adiponectin	Adiponectin promotes HO-1 induction by simultaneously activating Nrf2 and BRG1 to reduce cardiac oxidative stress, improve cardiac hypertrophy, and prevent left ventricular dysfunction in diabetic patients.	[39]
*β*-Adrenergic agonist	In vitro stimulation of myocardial cells with angiotensin II or a *β*-adrenergic agonist results in increased BRG1 expression.	[116]
CDK inhibitors	CDK9 inhibition dephosphorylates the SWI/SNF protein BRG1, which contributes to gene reactivation.	[127]
Darinaparsin	Darinaparsin inhibits HO-1 transcription by causing BRG1 phosphorylation through G2/M cell cycle arrest.	[33]
ENT	At the protein level, ENT reduces BRG1 protein abundance in Rh30 and U23674 cells, notably to an undetected level.	[128]
IsoPostC	Emulsified IsoPostC protects the heart through BRG1/Nrf2/STAT3 signaling.	[38]
NAC	The enhanced expression of BRG1 may be a new mechanism by which antioxidant NAC provides cardiac protection.	[36]
Oridonin	Oridonin inhibits proliferation of Jurkat cells via the downregulation of BRG1.	[129]
PFI-3	PFI-3 is a highly potent, selective, and cell-permeable inhibitor for the BRG1/BRM.	[130, 131]
Phosphoaminoglycosides	Preparations of phosphoaminoglycosides were identified as inhibitors of the in vitro activities of three SWI2/SNF2 family members.	[132]
PPC	PPC provides protection to H/R-induced L02 cells by activating Nrf2 and BRG1.	[41]
Propofol	Propofol alleviates oxidative stress in anoxia/reoxygenated hepatocytes by upregulating lncrna-TUG1/BRG1 pathway.	[40]
Rosiglitazone	The expression of BRG1 was significantly increased in cardiac remodeling heart, and the change can be reversed by rosiglitazone.	[124]
SPostC	SPostC prevents hypoxia-reoxygenation-induced cardiomyocyte damage and oxidative stress by activating Nrf2/BRG1/HO-1 signaling.	[37]
Tetrandrine	Tetrandrine upregulated BRG1 expression in a dose- and time-dependent pattern in Hep-2 cells.	[133]

## References

[1] Clapier C. R., Cairns B. R. (2009). The biology of chromatin remodeling complexes. *Annual Review of Biochemistry*.

[2] Trotter K. W., Archer T. K. (2007). Nuclear receptors and chromatin remodeling machinery. *Molecular and Cellular Endocrinology*.

[3] Mohrmann L., Verrijzer C. P. (2005). Composition and functional specificity of SWI2/SNF2 class chromatin remodeling complexes. *Biochimica et Biophysica Acta (BBA) - Gene Structure and Expression*.

[4] Wu Q., Madany P., Dobson J. R. (2016). The BRG1 chromatin remodeling enzyme links cancer cell metabolism and proliferation. *Oncotarget*.

[5] Singh A. P., Foley J. F., Rubino M. (2016). Brg1 enables rapid growth of the early embryo by suppressing genes that regulate apoptosis and cell growth arrest. *Molecular and Cellular Biology*.

[6] Marathe H. G., Watkins-Chow D. E., Weider M. (2017). BRG1 interacts with SOX10 to establish the melanocyte lineage and to promote differentiation. *Nucleic Acids Research*.

[7] Pyo J.-S., Son B. K., Oh D., Kim E. K. (2018). BRG1 is correlated with poor prognosis in colorectal cancer. *Human Pathology*.

[8] Li H., Lan J., Han C. (2018). Brg1 promotes liver fibrosis via activation of hepatic stellate cells. *Experimental Cell Research*.

[9] Vieira J. M., Howard S., Villa del Campo C. (2017). BRG1-SWI/SNF-dependent regulation of the Wt1 transcriptional landscape mediates epicardial activity during heart development and disease. *Nature Communications*.

[10] Menoni H., Gasparutto D., Hamiche A. (2007). ATP-dependent chromatin remodeling is required for base excision repair in conventional but not in variant H2A.Bbd nucleosomes. *Molecular and Cellular Biology*.

[11] Thorpe G. W., Fong C. S., Alic N., Higgins V. J., Dawes I. W. (2004). Cells have distinct mechanisms to maintain protection against different reactive oxygen species: oxidative-stress-response genes. *Proceedings of the National Academy of Sciences of the United States of America*.

[12] Freeman M. D., Mazu T., Miles J. S., Darling-Reed S., Flores-Rozas H. (2014). Inactivation of chromatin remodeling factors sensitizes cells to selective cytotoxic stress. *Biologics: Targets & Therapy*.

[13] Kim J.-M., To T. K., Nishioka T., Seki M. (2010). Chromatin regulation functions in plant abiotic stress responses. *Plant, Cell & Environment*.

[14] Medina P. P., Romero O. A., Kohno T. (2008). Frequent BRG1/SMARCA4-inactivating mutations in human lung cancer cell lines. *Human Mutation*.

[15] Du W., Rani R., Sipple J. (2012). The FA pathway counteracts oxidative stress through selective protection of antioxidant defense gene promoters. *Blood*.

[16] Sena J. A., Wang L., Hu C.-J. (2013). BRG1 and BRM chromatin-remodeling complexes regulate the hypoxia response by acting as coactivators for a subset of hypoxia-inducible transcription factor target genes. *Molecular and Cellular Biology*.

[17] Zhao J., du F., Shen G., Zheng F., Xu B. (2015). The role of hypoxia-inducible factor-2 in digestive system cancers. *Cell Death & Disease*.

[18] Nakazawa M. S., Keith B., Simon M. C. (2016). Oxygen availability and metabolic adaptations. *Nature Reviews Cancer*.

[19] Xia J., Jiang N., Li Y., Wei Y., Zhang X. (2019). The long noncoding RNA THRIL knockdown protects hypoxia-induced injuries of H9C2 cells through regulating miR-99a. *Cardiology Journal*.

[20] Valko M., Leibfritz D., Moncol J., Cronin M. T., Mazur M., Telser J. (2007). Free radicals and antioxidants in normal physiological functions and human disease. *The International Journal of Biochemistry & Cell Biology*.

[21] Bae Y. S., Oh H., Rhee S. G., Yoo Y. D. (2011). Regulation of reactive oxygen species generation in cell signaling. *Molecules and Cells*.

[22] Pruchniak M. P., Aražna M., Demkow U. (2016). Biochemistry of oxidative stress. *Advances in Experimental Medicine and Biology*.

[23] Weidinger A., Kozlov A. V. (2015). Biological activities of reactive oxygen and nitrogen species: oxidative stress versus signal transduction. *Biomolecules*.

[24] Sies H., Berndt C., Jones D. P. (2017). Oxidative stress. *Annual Review of Biochemistry*.

[25] Townsend D. M., Lushchak V. I., Cooper A. J. L. (2014). A comparison of reversible versus irreversible protein glutathionylation. *Advances in Cancer Research*.

[26] Feoli A. M. P., Macagnan F. E., Piovesan C. H., Bodanese L. C., Siqueira I. R. (2014). Xanthine oxidase activity is associated with risk factors for cardiovascular disease and inflammatory and oxidative status markers in metabolic syndrome: effects of a single exercise session. *Oxidative Medicine and Cellular Longevity*.

[27] Ahmad F., Nidadavolu P., Durgadoss L., Ravindranath V. (2014). Critical cysteines in Akt 1 regulate its activity and proteasomal degradation: implications for neurodegenerative diseases. *Free Radical Biology & Medicine*.

[28] Yan L.-J. (2014). Pathogenesis of chronic hyperglycemia: from reductive stress to oxidative stress. *Journal of Diabetes Research*.

[29] Cadet J., Douki T., Ravanat J.-L. (2010). Oxidatively generated base damage to cellular DNA. *Free Radical Biology & Medicine*.

[30] Klaunig J. E., Kamendulis L. M., Hocevar B. A. (2009). Oxidative stress and oxidative damage in carcinogenesis. *Toxicologic Pathology*.

[31] Lee J.-M., Johnson J. A. (2004). An important role of Nrf2-ARE pathway in the cellular defense mechanism. *Journal of Biochemistry and Molecular Biology*.

[32] Ishii T., Mann G. E. (2014). Redox status in mammalian cells and stem cells during culture in vitro: critical roles of Nrf 2 and cystine transporter activity in the maintenance of redox balance. *Redox Biology*.

[33] Garnier N., Petruccelli L. A., Molina M. F. (2013). The novel arsenical Darinaparsin circumvents BRG1-dependent, HO-1-mediated cytoprotection in leukemic cells. *Leukemia*.

[34] Zhang J., Ohta T., Maruyama A. (2006). BRG1 interacts with Nrf2 to selectively mediate HO-1 induction in response to oxidative stress. *Molecular and Cellular Biology*.

[35] Maruyama A., Mimura J., Harada N., Itoh K. (2013). Nrf2 activation is associated with Z-DNA formation in the human HO-1 promoter. *Nucleic Acids Research*.

[36] Xu J., Lei S., Liu Y. (2013). Antioxidant N-acetylcysteine attenuates the reduction of Brg1 protein expression in the myocardium of type 1 diabetic rats. *Journal of Diabetes Research*.

[37] Gao S., Yang Z., Shi R. (2016). Diabetes blocks the cardioprotective effects of sevoflurane postconditioning by impairing Nrf2/Brg1/HO-1 signaling. *European Journal of Pharmacology*.

[38] Wang Y., Li H., Huang H. (2016). Cardioprotection from emulsified isoflurane postconditioning is lost in rats with streptozotocin-induced diabetes due to the impairment of Brg1/Nrf2/STAT3 signalling. *Clinical Science*.

[39] Li H., Yao W., Irwin M. G. (2015). Adiponectin ameliorates hyperglycemia-induced cardiac hypertrophy and dysfunction by concomitantly activating Nrf2 and Brg1. *Free Radical Biology & Medicine*.

[40] Ming N., Na H. S. T., He J. L., Meng Q. T., Xia Z. Y. (2019). Propofol alleviates oxidative stress via upregulating lncRNA-TUG1/Brg1 pathway in hypoxia/reoxygenation hepatic cells. *Journal of Biochemistry*.

[41] Ge M., Chen H., Zhu Q. (2017). Propofol post-conditioning alleviates hepatic ischaemia reperfusion injury via BRG1-mediated Nrf2/HO-1 transcriptional activation in human and mice. *Journal of Cellular and Molecular Medicine*.

[42] Ge M., Yao W., Yuan D. (2017). Brg1-mediated Nrf2/HO-1 pathway activation alleviates hepatic ischemia-reperfusion injury. *Cell Death & Disease*.

[43] Ge M., Chen C., Yao W. (2017). Overexpression of Brg1 alleviates hepatic ischemia/reperfusion-induced acute lung injury through antioxidative stress effects. *Oxidative Medicine and Cellular Longevity*.

[44] Deng L., Li G., Rao B., Li H. (2015). Central nervous system-specific knockout of Brg1 causes growth retardation and neuronal degeneration. *Brain Research*.

[45] Li F., Liang J., Tang D. (2018). Brahma-related gene 1 ameliorates the neuronal apoptosis and oxidative stress induced by oxygen-glucose deprivation/reoxygenation through activation of Nrf2/HO-1 signaling. *Biomedicine & Pharmacotherapy*.

[46] Li Y., Zhao Y., Cheng M. (2018). Suppression of microRNA-144-3p attenuates oxygen-glucose deprivation/reoxygenation-induced neuronal injury by promoting Brg1/Nrf2/ARE signaling. *Journal of Biochemical and Molecular Toxicology*.

[47] Li F., Liang J., Tong H., Zhu S., Tang D. (2020). Inhibition of microRNA-199a-5p ameliorates oxygen-glucose deprivation/reoxygenation-induced apoptosis and oxidative stress in HT22 neurons by targeting Brg1 to activate Nrf2/HO-1 signalling. *Clinical and Experimental Pharmacology & Physiology*.

[48] Blaser H., Dostert C., Mak T. W., Brenner D. (2016). TNF and ROS crosstalk in inflammation. *Trends in Cell Biology*.

[49] Naito M., Zager R. A., Bomsztyk K. (2009). BRG1 increases transcription of proinflammatory genes in renal ischemia. *Journal of the American Society of Nephrology*.

[50] Liu L., Mao L., Wu X. (2019). BRG1 regulates endothelial-derived IL-33 to promote ischemia-reperfusion induced renal injury and fibrosis in mice. *Biochimica et Biophysica Acta-Molecular Basis of Disease*.

[51] Drenger B., Ostrovsky I. A., Barak M., Nechemia-Arbely Y., Ziv E., Axelrod J. H. (2011). Diabetes blockade of sevoflurane postconditioning is not restored by insulin in the rat heart: phosphorylated signal transducer and activator of transcription 3- and phosphatidylinositol 3-kinase-mediated inhibition. *Anesthesiology*.

[52] Fish J. E., Yan M. S., Matouk C. C. (2010). Hypoxic repression of endothelial nitric-oxide synthase transcription is coupled with eviction of promoter histones. *The Journal of Biological Chemistry*.

[53] Huang M., Qian F., Hu Y., Ang C., Li Z., Wen Z. (2002). Chromatin-remodelling factor BRG1 selectively activates a subset of interferon-alpha-inducible genes. *Nature Cell Biology*.

[54] Schoonbroodt S., Ferreira V., Best-Belpomme M. (2000). Crucial role of the amino-terminal tyrosine residue 42 and the carboxyl-terminal PEST domain of I kappa B alpha in NF-kappa B activation by an oxidative stress. *Journal of Immunology*.

[55] Takada Y., Mukhopadhyay A., Kundu G. C., Mahabeleshwar G. H., Singh S., Aggarwal B. B. (2003). Hydrogen peroxide activates NF-kappa B through tyrosine phosphorylation of I kappa B alpha and serine phosphorylation of p65: evidence for the involvement of I kappa B alpha kinase and Syk protein-tyrosine kinase. *The Journal of Biological Chemistry*.

[56] Stenmark K. R., Fagan K. A., Frid M. G. (2006). Hypoxia-induced pulmonary vascular remodeling: cellular and molecular mechanisms. *Circulation Research*.

[57] Tong Q., Zheng L., Lin L., Li B., Wang D., Li D. (2006). Hypoxia-induced mitogenic factor promotes vascular adhesion molecule-1 expression via the PI-3K/Akt-NF-kappaB signaling pathway. *American Journal of Respiratory Cell and Molecular Biology*.

[58] Fang F., Chen D., Yu L. (2013). Proinflammatory stimuli engage Brahma related gene 1 and Brahma in endothelial injury. *Circulation Research*.

[59] Chen Y., Liu K., Shi Y., Shao C. (2018). The tango of ROS and p53 in tissue stem cells. *Cell Death and Differentiation*.

[60] Lee S.-R., Yang K. S., Kwon J., Lee C., Jeong W., Rhee S. G. (2002). Reversible inactivation of the tumor suppressor PTEN by H2O2. *The Journal of Biological Chemistry*.

[61] Murata H., Ihara Y., Nakamura H., Yodoi J., Sumikawa K., Kondo T. (2003). Glutaredoxin exerts an antiapoptotic effect by regulating the redox state of Akt. *The Journal of Biological Chemistry*.

[62] Naidu S. R., Love I. M., Imbalzano A. N., Grossman S. R., Androphy E. J. (2009). The SWI/SNF chromatin remodeling subunit BRG1 is a critical regulator of p53 necessary for proliferation of malignant cells. *Oncogene*.

[63] Watanabe T., Semba S., Yokozaki H. (2011). Regulation of PTEN expression by the SWI/SNF chromatin-remodelling protein BRG1 in human colorectal carcinoma cells. *British Journal of Cancer*.

[64] Liu X., Tian X., Wang F., Ma Y., Kornmann M., Yang Y. (2014). BRG1 promotes chemoresistance of pancreatic cancer cells through crosstalking with Akt signalling. *European Journal of Cancer*.

[65] Chen K.-Y., Chiu C.-H., Wang L.-C. (2017). Anti-apoptotic effects of Sonic hedgehog signalling through oxidative stress reduction in astrocytes co-cultured with excretory-secretory products of larval Angiostrongylus cantonensis. *Scientific Reports*.

[66] Shi X., Wang Q., Gu J., Xuan Z., Wu J. I. (2016). SMARCA4/Brg1 coordinates genetic and epigenetic networks underlying Shh-type medulloblastoma development. *Oncogene*.

[67] Xiong Y., Li W., Shang C. (2013). Brg1 governs a positive feedback circuit in the hair follicle for tissue regeneration and repair. *Developmental Cell*.

[68] Liu R.-M., Desai L. P. (2015). Reciprocal regulation of TGF-*β* and reactive oxygen species: a perverse cycle for fibrosis. *Redox Biology*.

[69] Korswagen H. C. (2006). Regulation of the Wnt/beta-catenin pathway by redox signaling. *Developmental Cell*.

[70] Ross S., Cheung E., Petrakis T. G., Howell M., Kraus W. L., Hill C. S. (2006). Smads orchestrate specific histone modifications and chromatin remodeling to activate transcription. *The EMBO Journal*.

[71] Griffin C. T., Curtis C. D., Davis R. B., Muthukumar V., Magnuson T. (2011). The chromatin-remodeling enzyme BRG1 modulates vascular Wnt signaling at two levels. *Proceedings of the National Academy of Sciences of the United States of America*.

[72] Li N., Kong M., Zeng S. (2018). Brahma related gene 1 (Brg1) contributes to liver regeneration by epigenetically activating the Wnt/*β*-catenin pathway in mice. *The FASEB Journal*.

[73] Skulachev V. P. (2012). Mitochondria-targeted antioxidants as promising drugs for treatment of age-related brain diseases. *Journal of Alzheimer's Disease*.

[74] Yi D.-G., Hong S., Huh W.-K. (2018). Mitochondrial dysfunction reduces yeast replicative lifespan by elevating RAS-dependent ROS production by the ER-localized NADPH oxidase Yno 1. *PLoS One*.

[75] Tan K., Fujimoto M., Takii R., Takaki E., Hayashida N., Nakai A. (2015). Mitochondrial SSBP1 protects cells from proteotoxic stresses by potentiating stress-induced HSF1 transcriptional activity. *Nature Communications*.

[76] Bultman S. J., Holley D. W., G de Ridder G. (2016). BRG1 and BRM SWI/SNF ATPases redundantly maintain cardiomyocyte homeostasis by regulating cardiomyocyte mitophagy and mitochondrial dynamics in vivo. *Cardiovascular Pathology*.

[77] Lissanu Deribe Y., Sun Y., Terranova C. (2018). Mutations in the SWI/SNF complex induce a targetable dependence on oxidative phosphorylation in lung cancer. *Nature Medicine*.

[78] Huang X., Chen X., He Y. (2017). Mitochondrial complex I bridges a connection between regulation of carbon flexibility and gastrointestinal commensalism in the human fungal pathogen Candida albicans. *PLoS Pathogens*.

[79] Dutta A., Sardiu M., Gogol M. (2017). Composition and function of mutant Swi/Snf complexes. *Cell Reports*.

[80] Appenzeller-Herzog C. (2012). Updates on "endoplasmic reticulum redox". *Antioxidants & Redox Signaling*.

[81] Ron D., Walter P. (2007). Signal integration in the endoplasmic reticulum unfolded protein response. *Nature Reviews Molecular Cell Biology*.

[82] Sahu R. K., Saha N., Das L., Sahu P. K., Sariki S. K., Tomar R. S. (2020). SWI/SNF chromatin remodelling complex contributes to clearance of cytoplasmic protein aggregates and regulates unfolded protein response in *Saccharomyces cerevisiae*. *The FEBS Journal*.

[83] Levine B., Kroemer G. (2008). Autophagy in the pathogenesis of disease. *Cell*.

[84] Cadwell K., Liu J. Y., Brown S. L. (2008). A key role for autophagy and the autophagy gene Atg16l1 in mouse and human intestinal Paneth cells. *Nature*.

[85] Patel K. K., Miyoshi H., Beatty W. L. (2013). Autophagy proteins control goblet cell function by potentiating reactive oxygen species production. *The EMBO Journal*.

[86] Liu M., Sun T., Li N. (2019). BRG1 attenuates colonic inflammation and tumorigenesis through autophagy-dependent oxidative stress sequestration. *Nature Communications*.

[87] Brandes R. P., Weissmann N., Schröder K. (2014). Redox-mediated signal transduction by cardiovascular Nox NADPH oxidases. *Journal of Molecular and Cellular Cardiology*.

[88] Nayernia Z., Jaquet V., Krause K.-H. (2014). New insights on NOX enzymes in the central nervous system. *Antioxidants & Redox Signaling*.

[89] Briggs R. T., Karnovsky M. L., Karnovsky M. J. (1977). Hydrogen peroxide production in chronic granulomatous disease. A cytochemical study of reduced pyridine nucleotide oxidases. *The Journal of Clinical Investigation*.

[90] Bedard K., Krause K.-H. (2007). The NOX family of ROS-generating NADPH oxidases: physiology and pathophysiology. *Physiological Reviews*.

[91] Hecker L., Vittal R., Jones T. (2009). NADPH oxidase-4 mediates myofibroblast activation and fibrogenic responses to lung injury. *Nature Medicine*.

[92] Li Z., Zhang X., Liu S. (2018). BRG1 regulates NOX gene transcription in endothelial cells and contributes to cardiac ischemia-reperfusion injury. *Biochimica et Biophysica Acta-Molecular Basis of Disease*.

[93] Liu L., Hong W., Li M. (2019). A cross talk between BRG1 and males absent on the first contributes to reactive oxygen species production in a mouse model of nonalcoholic steatohepatitis. *Antioxidants & Redox Signaling*.

[94] Li Z., Chen B., Dong W. (2019). The chromatin remodeler Brg1 integrates ROS production and endothelial-mesenchymal transition to promote liver fibrosis in mice. *Frontiers in Cell and Developmental Biology*.

[95] Bian K., Murad F. (2003). Nitric oxide (NO)–biogeneration, regulation, and relevance to human diseases. *Frontiers in Bioscience*.

[96] Förstermann U., Sessa W. C. (2012). Nitric oxide synthases: regulation and function. *European Heart Journal*.

[97] Förstermann U., Münzel T. (2006). Endothelial nitric oxide synthase in vascular disease: from marvel to menace. *Circulation*.

[98] Shao J., Xu Y., Fang M. (2020). BRG1 deficiency in endothelial cells alleviates thioacetamide induced liver fibrosis in mice. *Biochemical and Biophysical Research Communications*.

[99] Cherian M. G., Jayasurya A., Bay B.-H. (2003). Metallothioneins in human tumors and potential roles in carcinogenesis. *Mutation Research*.

[100] You H. J., Lee K. J., Jeong H. G. (2002). Overexpression of human metallothionein-III prevents hydrogen peroxide-induced oxidative stress in human fibroblasts. *FEBS Letters*.

[101] Datta J., Majumder S., Bai S. (2005). Physical and functional interaction of DNA methyltransferase 3A with Mbd3 and Brg1 in mouse lymphosarcoma cells. *Cancer Research*.

[102] Wood G. E., Khelef N., Guiso N., Friedman R. L. (1998). Identification of Btr-regulated genes using a titration assay. Search for a role for this transcriptional regulator in the growth and virulence of Bordetella pertussis. *Gene*.

[103] Cajigas I., Leib D. E., Cochrane J. (2015). Evf2 lncRNA/BRG1/DLX1 interactions reveal RNA-dependent inhibition of chromatin remodeling. *Development*.

[104] Wang X., Gong Y., Jin B. (2014). Long non-coding RNA urothelial carcinoma associated 1 induces cell replication by inhibiting BRG1 in 5637 cells. *Oncology Reports*.

[105] Coira I. F., Rufino-Palomares E. E., Romero O. A. (2015). Expression inactivation of SMARCA4 by microRNAs in lung tumors. *Human Molecular Genetics*.

[106] Chang Y., Cui M., Fu X. (2019). MiRNA-155 regulates lymphangiogenesis in natural killer/T-cell lymphoma by targeting BRG1. *Cancer Biology & Therapy*.

[107] Cuadros M., Sánchez-Martín V., Herrera A. (2017). BRG1 regulation by miR-155 in human leukemia and lymphoma cell lines. *Clinical & Translational Oncology*.

[108] Zhang H., Sun Z., Yu L., Sun J. (2017). MiR-139-5p inhibits proliferation and promoted apoptosis of human airway smooth muscle cells by downregulating the Brg1 gene. *Respiratory Physiology & Neurobiology*.

[109] Choi J., Kim Y. K., Park K. (2016). MicroRNA-139-5p regulates proliferation of hematopoietic progenitors and is repressed during BCR-ABL-mediated leukemogenesis. *Blood*.

[110] Möbus S., Yang D., Yuan Q. (2015). MicroRNA-199a-5p inhibition enhances the liver repopulation ability of human embryonic stem cell-derived hepatic cells. *Journal of Hepatology*.

[111] Schramedei K., Mörbt N., Pfeifer G. (2011). MicroRNA-21 targets tumor suppressor genes ANP32A and SMARCA4. *Oncogene*.

[112] Seeley J. J., Baker R. G., Mohamed G. (2018). Induction of innate immune memory via microRNA targeting of chromatin remodelling factors. *Nature*.

[113] Cui Y., Xie S., Luan J., Zhou X., Han J. (2013). Quantitative proteomics and protein network analysis of A549 lung cancer cells affected by miR-206. *Bioscience Trends*.

[114] Wade S. L., Langer L. F., Ward J. M., Archer T. K. (2015). MiRNA-mediated regulation of the SWI/SNF chromatin remodeling complex controls pluripotency and endodermal differentiation in human ESCs. *Stem Cells*.

[115] Jégu T., Blum R., Cochrane J. C. (2019). Xist RNA antagonizes the SWI/SNF chromatin remodeler BRG1 on the inactive X chromosome. *Nature Structural & Molecular Biology*.

[116] Mehta G., Kumarasamy S., Wu J. (2015). MITF interacts with the SWI/SNF subunit, BRG1, to promote GATA4 expression in cardiac hypertrophy. *Journal of Molecular and Cellular Cardiology*.

[117] Witwicka H., Nogami J., Syed S. A. (2019). Calcineurin broadly regulates the initiation of skeletal muscle-specific gene expression by binding target promoters and facilitating the interaction of the SWI/SNF chromatin remodeling enzyme. *Molecular and Cellular Biology*.

[118] Cao M., Wu J. I. (2015). Camk2a-Cre-mediated conditional deletion of chromatin remodeler Brg1 causes perinatal hydrocephalus. *Neuroscience Letters*.

[119] Padilla-Benavides T., Nasipak B. T., Paskavitz A. L. (2017). Casein kinase 2-mediated phosphorylation of Brahma-related gene 1 controls myoblast proliferation and contributes to SWI/SNF complex composition. *The Journal of Biological Chemistry*.

[120] Nguyen T. T., Savory J. G., Brooke-Bisschop T. (2017). Cdx2 regulates gene expression through recruitment of Brg1-associated switch-sucrose non-fermentable (SWI-SNF) chromatin remodeling activity. *The Journal of Biological Chemistry*.

[121] Kim J.-H., Saraf A., Florens L., Washburn M., Workman J. L. (2010). Gcn5 regulates the dissociation of SWI/SNF from chromatin by acetylation of Swi2/Snf2. *Genes & Development*.

[122] Cleary I. A., Lazzell A. L., Monteagudo C., Thomas D. P., Saville S. P. (2012). BRG1 and NRG1 form a novel feedback circuit regulating Candida albicans hypha formation and virulence. *Molecular Microbiology*.

[123] Mardaryev A. N., Gdula M. R., Yarker J. L. (2013). p63 and Brg1 control developmentally regulated higher-order chromatin remodelling at the epidermal differentiation complex locus in epidermal progenitor cells. *Development*.

[124] Qi H.-P., Wang Y., Zhang Q. H. (2015). Activation of peroxisome proliferator-activated receptor *γ* (PPAR*γ*) through NF-*κ*B/Brg1 and TGF-*β*1 pathways attenuates cardiac remodeling in pressure-overloaded rat hearts. *Cellular Physiology and Biochemistry*.

[125] Huang L.-Y., Zhao J., Chen H. (2018). SCF^FBW7^-mediated degradation of Brg1 suppresses gastric cancer metastasis. *Nature Communications*.

[126] Sohn D. H., Lee K. Y., Lee C. (2007). SRG3 interacts directly with the major components of the SWI/SNF chromatin remodeling complex and protects them from proteasomal degradation. *The Journal of Biological Chemistry*.

[127] Zhang H., Pandey S., Travers M. (2018). Targeting CDK9 reactivates epigenetically silenced genes in cancer. *Cell*.

[128] Bharathy N., Berlow N. E., Wang E. (2018). The HDAC3-SMARCA4-miR-27a axis promotes expression of thePAX3:FOXO1fusion oncogene in rhabdomyosarcoma. *Science Signaling*.

[129] Ye Z.-Z., Xue F. L., Ding W. P., Kong X., Shen Y. N. (2017). Oridonin inhibits proliferation of Jurkat cells via the down-regulation of Brg1. *Zhongguo dang dai er ke za zhi = Chinese Journal of Contemporary Pediatrics*.

[130] Vangamudi B., Paul T. A., Shah P. K. (2015). The SMARCA2/4 ATPase domain surpasses the bromodomain as a drug target in SWI/SNF-mutant cancers: insights from cDNA rescue and PFI-3 inhibitor studies. *Cancer Research*.

[131] Fedorov O., Castex J., Tallant C. (2015). Selective targeting of the BRG/PB1 bromodomains impairs embryonic and trophoblast stem cell maintenance. *Science Advances*.

[132] Muthuswami R., Mesner L. D., Wang D., Hill D. A., Imbalzano A. N., Hockensmith J. W. (2000). Phosphoaminoglycosides inhibit SWI2/SNF2 family DNA-dependent molecular motor domains. *Biochemistry*.

[133] Cui X., Zhu W., Wang P., Wang X. (2015). Tetrandrine inhibits the intracellular calcium ion level and upregulates the expression of Brg1 and AHNAK in Hep-2 cells. *Clinical Laboratory*.

